# Nail Whispers Revealing Dermatological and Systemic Secrets: An Analysis of Nail Disorders Associated With Diverse Dermatological and Systemic Conditions

**DOI:** 10.7759/cureus.45007

**Published:** 2023-09-11

**Authors:** Mansi Satasia, Amita H Sutaria

**Affiliations:** 1 Dermatology, Venereology and Leprology, B.J. Medical College and Civil Hospital, Ahmedabad, IND

**Keywords:** onychomycosis, epidemiology, dermoscopy, connective tissues disease, trachyonychia, nail psoriasis, nail diseases

## Abstract

Background and objective

Nail disorders encompass a wide spectrum of conditions, spanning congenital, developmental, infectious, neoplastic, degenerative, dermatological, and systemic diseases. A comprehensive exploration of their clinical manifestations, incidence, and associations is crucial for precise diagnosis and effective management.

Methods

This observational cross-sectional study conducted at B.J. Medical College and Civil Hospital, Ahmedabad involved 300 consecutive patients with nail changes from July 2017 to June 2019 reporting diverse dermatological and systemic conditions. The inclusion criteria involved patients of both genders and all age groups displaying nail changes associated with dermatological and systemic diseases. Data collection entailed a comprehensive clinical history, systemic and dermatological examinations, nail assessment using Dermoscope (DermLite 3, 10x), and supplementary tests. Analyses were performed on Microsoft Excel 2007 software. The study was approved by the Institute Ethics Committee.

Results

Among the 300 cases, females had a higher prevalence of nail involvement (57%), with a female-to-male ratio of 1.3:1. The most affected age group was 21-40 years, with 6-10 nails typically affected. Notably, housewives showed a higher prevalence. The most frequent nail condition was onychomycosis (24.33%) followed by psoriatic nail changes (20%). Less frequent nail changes involved eczema (5.7%), paronychia (5%), drug-induced (4.3%), lichen planus (3.7%), trauma-induced (3%), twenty nail dystrophy (2.33%), Darier's disease (2%), pemphigus vulgaris (2%), alopecia areata (1.67%), median Heller dystrophy (1.33%), atopic dermatitis (1%), epidermolysis bullosa (1%), racquet nail (1%), leprosy (1%), pityriasis rubra pilaris (0.67%), vitiligo (0.67%), secondary syphilis (0.67%), pachyonychia congenita (0.67%), as well as a case each of total leukonychia, subungual warts, Koenen tumor, and periungual fibroma(0.33%). Systemic autoimmune connective tissue disorders (CTD) accounted for 9%; the most common nail finding observed was nail fold erythema (48.1%) followed by nail fold telangiectasis (44.4%). In systemic sclerosis (SS), the most common finding was nail fold telangiectasia, and in systemic lupus erythematosus (SLE), the most common was nail fold erythema. Scleroderma capillary pattern on nail fold capillaroscopy was found in seven patients with SS, two patients with dermatomyositis, and only one patient with SLE. Nail changes observed in systemic diseases include onychomycosis in diabetes mellitus and chronic renal failure patients, splinter hemorrhages in ischemic heart disease and hypertension, longitudinal melanonychia in HIV, and koilonychia and platynychia in iron deficiency anemia. Other systemic diseases, such as Addison's disease and renal failure, also exhibited various nail changes.

Conclusions

Beyond their cosmetic importance, nails hold a vital pathologic role. Proficiency in nail terminology and classification is key for skillful evaluation. Understanding normal and abnormal nail variants, along with their disease associations, benefits diagnosis and tailored management. Nails, often overlooked but accessible, serve as a window into patients' general health and should be an integral part of thorough examinations. This study highlights an intricate clinical panorama of nail disorders, highlighting their significant role in both dermatological and systemic contexts.

## Introduction

Nails, like faces, reflect our internal and external health. Nails are tough, functional, and aesthetic. Nail disorders cause discomfort, impairing their function. Nail problems affect various parts of the body, influenced by genes, skin, infections, systemic diseases, aging, medications, trauma, and tumors. Dermatological conditions affecting skin and hair may impact nails.^ ^Around 10% of dermatologic cases affect nails [1]. Subtle nail changes signal systemic issues. In the fifth century, Hippocrates highlighted clubbing's significance in systemic manifestations [2]. Subsequently, various nail findings linked to systemic diseases have been identified. Abnormal nails are vital clinical clues, especially if they are unique. Recognizing nail changes aids in diagnosis. Nail assessment is crucial in dermatological exams, with fingernails offering clearer insights. Research on nail issues is limited, which, we believe, makes this study significant for understanding nail problems and associations.

## Materials and methods

Study type and period

This study followed an observational cross-sectional design and was conducted at the Dermatology Department of B.J. Medical College and Civil Hospital, spanning the period from July 2017 to June 2019.

Subject selection

The study included consecutive patients presenting with nail abnormalities associated with dermatological and systemic conditions. Participants of all ages and genders were considered, while those unwilling to provide consent were excluded.

Study design

All consecutive patients with nail changes presenting to the Dermatology Department from July 2017 to June 2019, spanning various ages and dermatological as well as systemic conditions, were included after obtaining informed consent. The sample size was determined by the formula n = Z^2^P(1−P) / d^2 ^where n is the sample size, Z is the statistic corresponding to the level of confidence, P is expected prevalence, considering the value for Z is 1.96, d is 4%, and P is 15%. Detailed clinical histories were gathered, accompanied by thorough systemic and dermatological examinations, all meticulously recorded. Nail examinations were conducted using a Dermoscope, with photographs taken for documentation. Routine investigations, microorganism cultures, Tzanck smears, fungus scrapings, nail clippings, capillaroscopy, and biopsies were performed as required. Data were meticulously entered into a specially designed proforma and subsequently analyzed using Microsoft Excel 2007 software.

## Results

Nail changes, a common manifestation of various dermatological and systemic conditions, hold significant clinical importance. This study aims to comprehensively analyze 300 consecutive cases presenting with nail changes to the Department of Dermatology and Venereology at Civil Hospital, Ahmedabad. The demographic profile (Table 1) of the participants revealed that among the 300 cases, 43% were male and 57% were female, with a female-to-male ratio of 1.3:1. The age range varied from three to 75 years, with a mean age of 35.2 years. The majority of patients with nail changes (42%) were in the age group of 21-40 years, followed by 28% in the age group of 41-60 years, 22% in the group of less than 20 years, and 8% over 60 years. The occupational distribution revealed that 46% of patients were housewives, 14% were in service/business, 19% were students, and 21% were laborers or farmers. Regarding the number of nails involved, 42.33% of cases had 6-10 nails affected, 38% had one to five nails involved, 11.67% had 11-15 nails affected, and 8% had 16-20 nails involved.

**Table 1:** Demographic characteristics of patients with nail changes (n=300)

Demographic characteristics		
Gender	Number of cases	Percentage
Male	129	43%
Female	171	57%
Age group, years	Number of cases	Percentage
<20	66	22%
21–40	126	42%
41–60	84	28%
>60	24	8%
Occupational status	Number of cases	Percentage
Housewife	138	46%
Students	57	19%
Laborer/farmer	63	21%
Service/business	42	14%
Number of nails involved	Number of cases	Percentage
1–5	114	38%
6–10	126	42%
11–15	36	12%
16–20	24	8%

Our study identified various dermatoses associated with nail changes (Table 2). Among the cases, 24.33% had onychomycosis, 20% had nail psoriasis, 5.7% had eczema, 5% had paronychia, and 4.3% were drug-induced. Lichen planus accounted for 3.7% of cases, followed by Darier's disease, pemphigus vulgaris, and twenty nail dystrophy, each at 2.33%. Alopecia areata, trauma-induced, median Heller dystrophy, and atopic dermatitis were among the less frequent cases.

**Table 2:** Nail changes in various dermatoses

Dermatoses	Number of cases	Percentage
Onychomycosis	73	24.33%
Psoriasis	60	20.00%
Lichen planus	11	3.70%
Paronychia	15	5.00%
Eczema	17	5.70%
Alopecia areata	5	1.70%
Drug-induced	13	4.30%
Periungual warts	1	0.33%
Atopic dermatitis	3	1.00%
Twenty nail dystrophy	7	2.33%
Nail changes due to trauma	9	3.00%
Secondary syphilis	2	1.06%
Leprosy	3	1.00%
Pemphigus vulgaris	6	2.00%
Vitiligo	2	0.67%
Darier’s disease	6	2.00%
Pachyonychia congenita	2	0.67%
Pityriasis rubra pilaris	2	0.67%
Epidermolysis bullosa	3	1.00%
Median Heller dystrophy	4	1.33%
Total idiopathic leukonychia	1	0.33%
Racquet nail	3	1.00%
Periungual fibroma	1	0.33%

**Figure 1:**
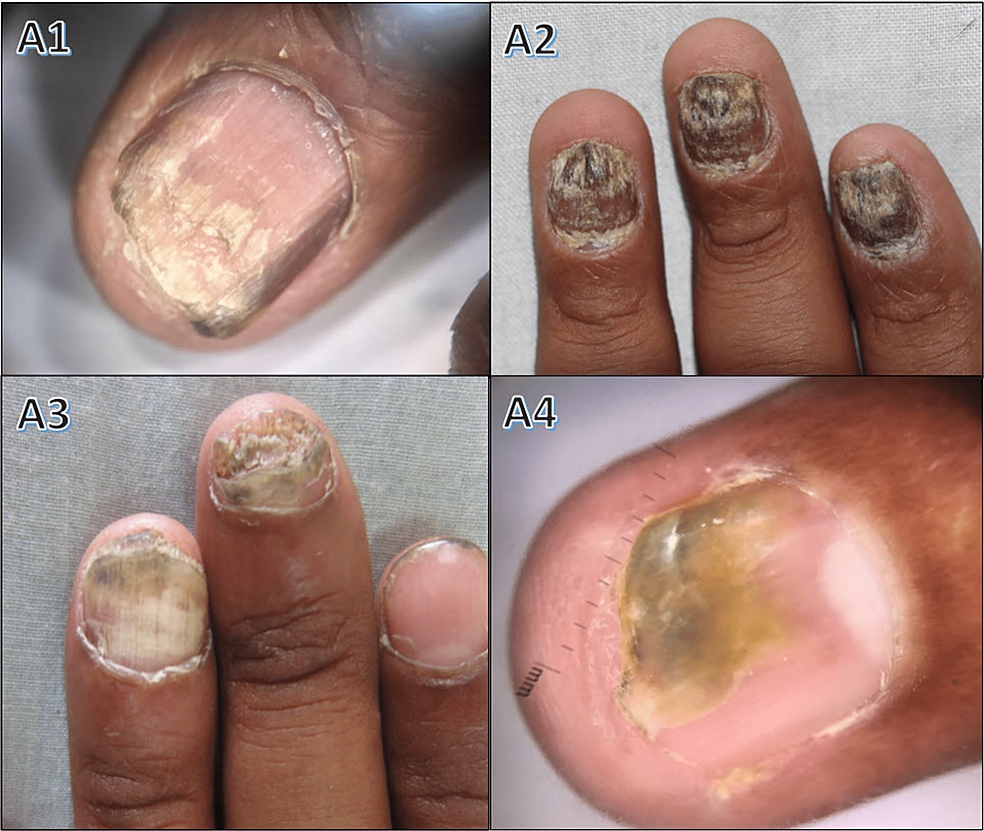
Morphological patterns of onychomycosis A1: superficial white onychomycosis (SWO); A2: total dystrophic onychomycosis (TDO); A3: proximal subungual onychomycosis (PSO); A4: distal lateral subungual onychomycosis (DLSO)

Specific dermatoses findings included onychomycosis, the most common nail disorder (24.33%), predominantly featuring distal lateral subungual onychomycosis (DLSO; 65.7%). Total dystrophic onychomycosis (TDO) and superficial white onychomycosis (SWO) were observed in 12.32% and 15.1% of cases, respectively, while proximal subungual onychomycosis (PSO) accounted for 6.8%, as shown in Table 3 and Figure 1.

**Table 3:** Demographics and subtype distribution of onychomycosis

Demographics and subtype distribution with regard to onychomycosis		
Age group, years	Male-to-female (number of cases)	Total number of cases (%)
<20	7:01	8 (10.96%)
21–40	18:16	34 (46.58%)
41–60	5:19	24 (32.88%)
>60	1:06	7 (9.59%)
Total	31:42:00	73 (100%)
Subtype of onychomycosis	Number of cases	Percentage
Distal lateral subungual onychomycosis (DLSO)	48	65.70%
Superficial white onychomycosis (SWO)	11	15.10%
Proximal subungual onychomycosis (PSO)	5	6.80%
Total dystrophic onychomycosis (TDO)	9	12.32%

**Figure 2:**
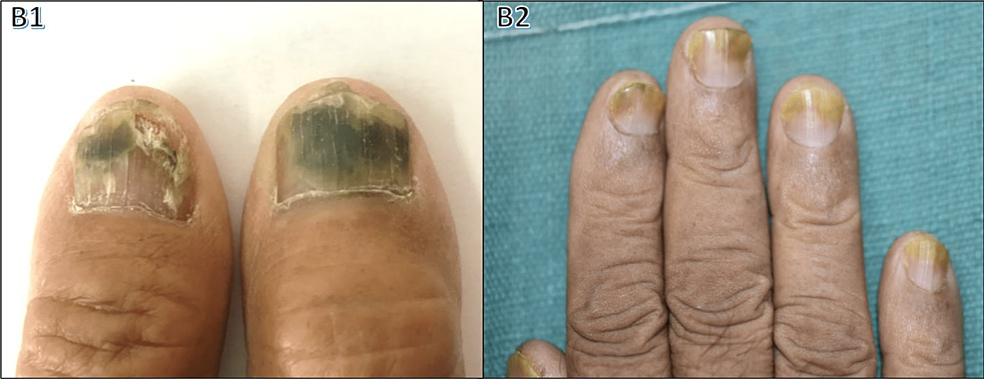
Nail plate discoloration in onychomycosis

Among onychomycosis patients, all displayed nail plate discoloration (100%); 68.5% had onychodystrophy (Figure 2), and 36.9% exhibited periungual erythema. Additionally, six onychomycosis patients had paronychia, three had diabetes mellitus, three had chronic renal failure, one had HIV on HAART, and one had pachyonychia congenita.

**Figure 3:**
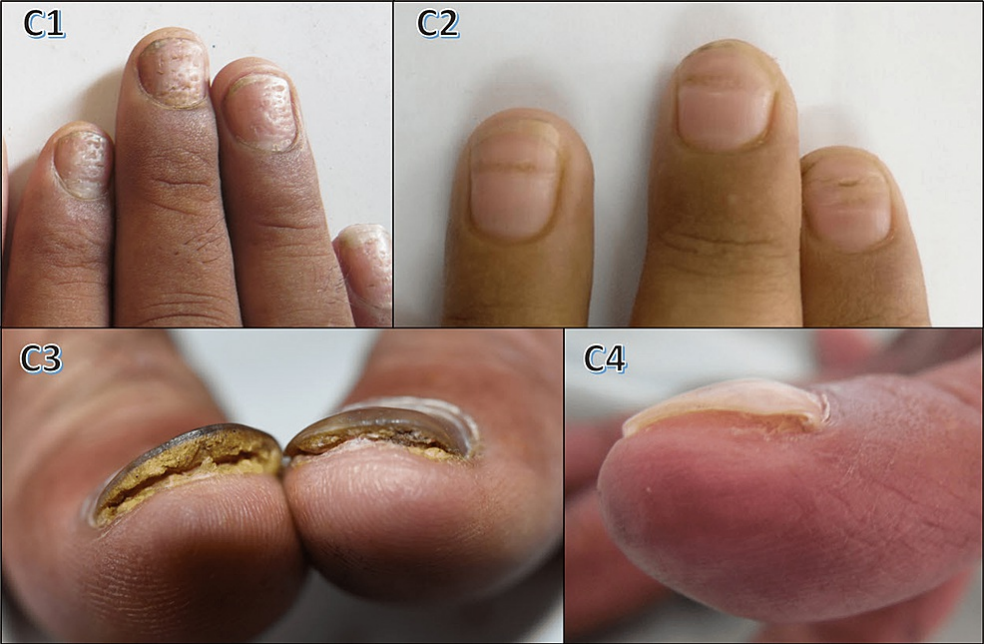
Nail changes in psoriasis C1: pitting; C2: Beau's lines; C3: subungual hyperkeratosis; C4: onycholysis

Psoriasis, the second most prevalent nail condition (20%), showed a male-to-female ratio of 2.1:1. The participants' age in this regard ranged from 14 to 69 years, with a mean age of 49.6 years (Table 4). Nail changes encompassed pitting (88.3%), onycholysis (56.7%), and subungual hyperkeratosis (43.3%), along with other features like discoloration, paronychia, splinter hemorrhages, Beau's lines, salmon patches, longitudinal ridging, and twenty nail dystrophy (Figure 3). Associated psoriatic arthritis was seen in seven patients, two psoriasis patients had associated diabetes mellitus, five patients suffered from systemic hypertension, and three had a history of atopy.

**Table 4:** Demographics and nail changes distribution in psoriasis

Demographics and nail changes distribution with regard to psoriasis		
Age group, years	Male-to-female (number of cases)	Total number of cases (%)
<20	3:01	4 (6.67%)
21–40	12:04	16 (26.67%)
41–60	21:11	32 (53.33%)
>60	5:03	8 (13.33%)
Total	41:19:00	60 (100%)
Nail changes in psoriasis	Number of cases	Percentage
Pitting	53	88.30%
Subungual hyperkeratosis	26	43.30%
Onycholysis	34	56.60%
Discoloration	11	18.30%
Paronychia	5	8.30%
Longitudinal ridging	13	21.70%
Beau's line	20	33.30%
Salmon patches	7	11.70%
Splinter hemorrhages	2	3.30%
Dystrophy	6	10.00%
Twenty nail dystrophy	1	1.70%

**Figure 4:**
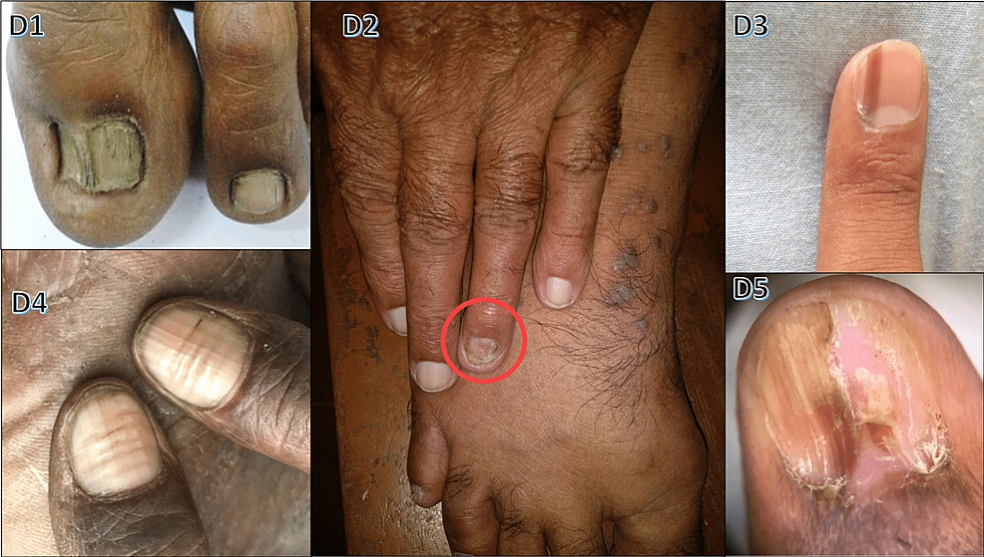
Nail changes in lichen planus D1: pterygium in toe nail; D2: onychodystrophy with lichen planus skin lesions; D3 and D4: Beau's lines and longitudinal ridging; D5: pterygium under dermoscopy

Lichen planus demonstrated longitudinal ridging (63.6%), thinning of the nail plate (54.5%), and longitudinal melanonychia (45.4%). Demographic characteristics and nail changes with respect to this condition are shown in Table 5 and Figure 4.

**Table 5:** Demographics and nail changes in lichen planus

Demographics and nail changes with regard to lichen planus		
Age group, years	Male-to-female (number of cases)	Total number of cases (%)
<20	1:01	2 (18.18% )
21–40	3:02	5 (45.45% )
41–60	2:01	3 (27.27% )
>60	0:01	1 (9.09% )
Total	6:05	11 (100% )
Nail changes in lichen planus	Number of cases	Percentage
Thinning of the nail plate	6	54.50%
Pterygium	3	27.20%
Longitudinal melanonychia	5	45.40%
Longitudinal ridging	7	63.60%
Beau’s lines	4	36.40%
Dystrophy	2	18.20%
Subungual hyperkeratosis	2	18.20%
Twenty nail dystrophy	1	9.10%

**Figure 5:**
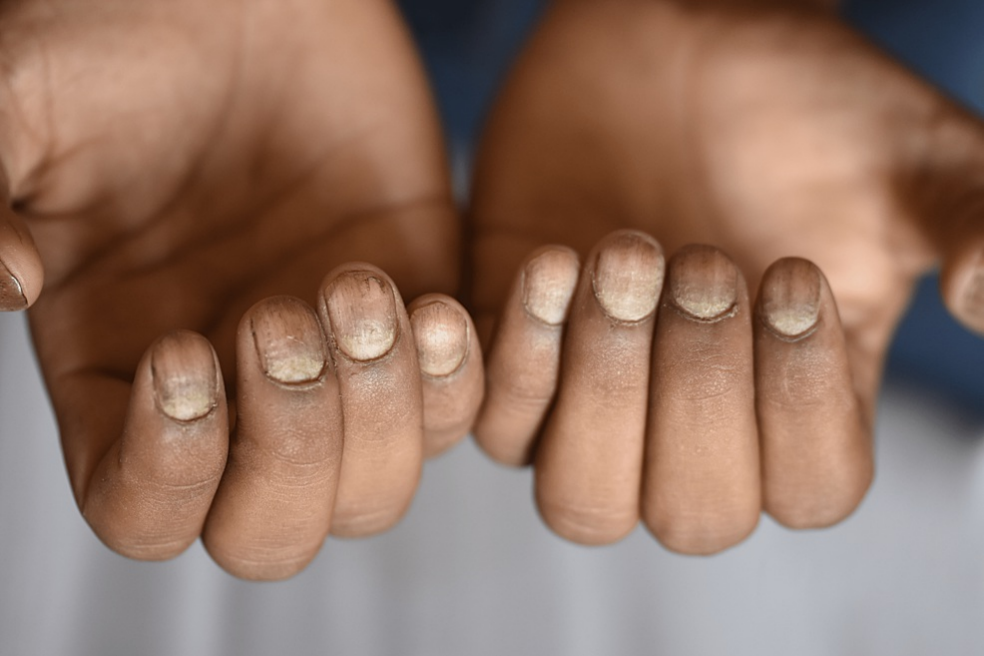
Trachyonychia (twenty nail dystrophy) seen in a patient with alopecia areata

Twenty nail dystrophy (Figure 5) was seen in 7 cases (2.33%). Out of the total seven cases, three were idiopathic, two involved alopecia areata, and there was one case each of psoriasis and lichen planus.

**Figure 6:**
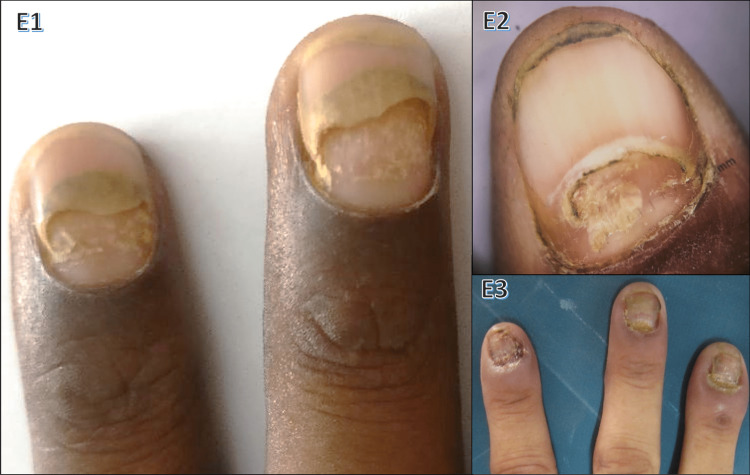
Nail changes in pemphigus vulgaris E1: onychomadesis; E2: dermoscopic image of onychomadesis; E3: paronychia, onychodystrophy

Among the six cases (2%) of pemphigus vulgaris, paronychia (66.67%) was the most common finding followed by onychomadesis and longitudinal ridges (50%) each. Nail changes in pemphigus are shown in Figure 6.

Various systemic diseases associated with nail changes are shown in Table 6.

**Table 6:** Nail changes associated with systemic diseases HIV: human immunodeficiency virus; HAART: highly active antiretroviral therapy; COPD: chronic obstructive pulmonary disease

Systemic diseases	Number of cases (N=300)	Percentage
Diabetes mellitus	4	1.33%
Chronic renal failure	4	1.33%
Ischemic heart disease and hypertension	2	0.67%
HIV on HAART	3	1.00%
Iron deficiency anemia	6	2.00%
Connective tissue disease	27	9.00%
COPD	1	0.33%
Addison’s disease	1	0.33%
Idiopathic	3	1.00%

**Figure 7:**
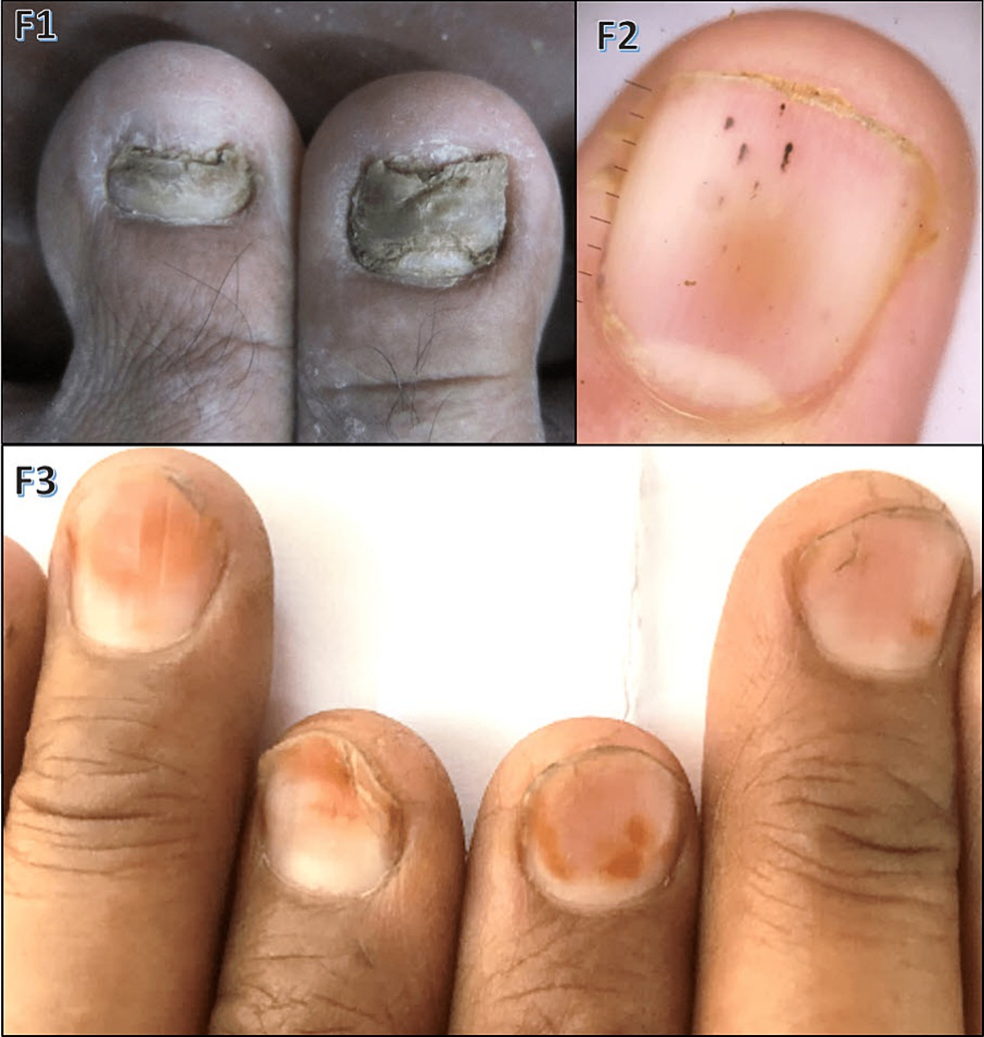
Various nail findings in systemic diseases F1: onychomycosis in a diabetic patient; F2: splinter hemorrhages in the dermoscopic image of the fingernail in a patient with hypertension and ischemic heart disease; F3: koilonychia in a patient with anemia

Among the observed cases, onychomycosis was seen in diabetes mellitus (Figure 7, F1) and chronic renal failure patients; Mee's lines and half-and-half nails were seen in chronic renal failure; splinter hemorrhages (Figure 7, F2) were seen in ischemic heart disease and hypertension, longitudinal melanonychia in HIV, and koilonychia (Figure 7, F3) and platynychia in iron deficiency anemia.

**Figure 8:**
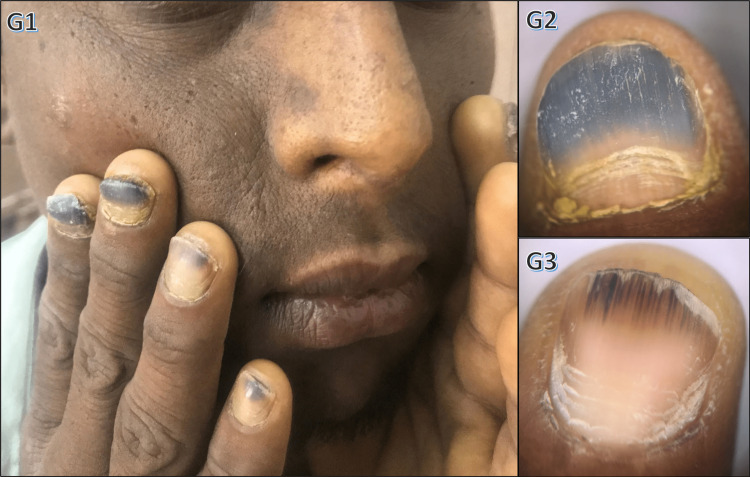
Nail changes in Addison's disease G1: nail plate discoloration and facial hyperpigmentation in a patient with Addison's disease; G2, G3: dermoscopic images of the same patient showing nail plate discoloration

Other systemic diseases, such as chronic obstructive pulmonary disease and Addison's disease (Figure 8), also exhibited specific nail changes.

Nail changes in connective tissue disorders (Table 7) were present in 27 patients (9%), including systemic lupus erythematosus (SLE), systemic sclerosis (SS), dermatomyositis (DMS), and mixed connective tissue diseases (MCTD). The most common nail finding observed was nail fold erythema(48.1%) followed by nail fold telangiectasis (44.4%), ragged cuticle (33.3%), and longitudinal ridging (33.3%). Scleroderma capillary pattern on NFC (reduced number of capillaries, severe avascularity, giant capillaries, and hemorrhage) was found in seven patients with SS, two patients with DMS, and only one patient with SLE.

**Table 7:** Nail unit changes in connective tissue disorders SS: systemic sclerosis; SLE: systemic lupus erythematosus; DMS: dermatomyositis; MCTD: mixed connective tissue diseases

Nail unit findings	SS (n=10)	SLE (n=13)	DMS (n=3)	MCTD (n=1)	Total (n=27)
Nail fold erythema	2	8	3	-	13 (48.1%)
Nail fold telangiectasia	8	2	1	1	12 (44.4%)
Ragged cuticle	5	3	1	-	9 (33.3%)
Splinter hemorrhages	1	-	-	-	1 (3.7%)
Longitudinal ridging	4	5	-	-	9 (33.3%)
Pseudoclubbing	1	-	-	-	1 (3.7%)
Red lunula	-	2	-	-	2 (7.4%)
Scleroderma capillary pattern	7	1	2	-	10 (37%)
H/o Raynaud’s phenomenon	10	2	-	1	13 (48.1%)

**Figure 9:**
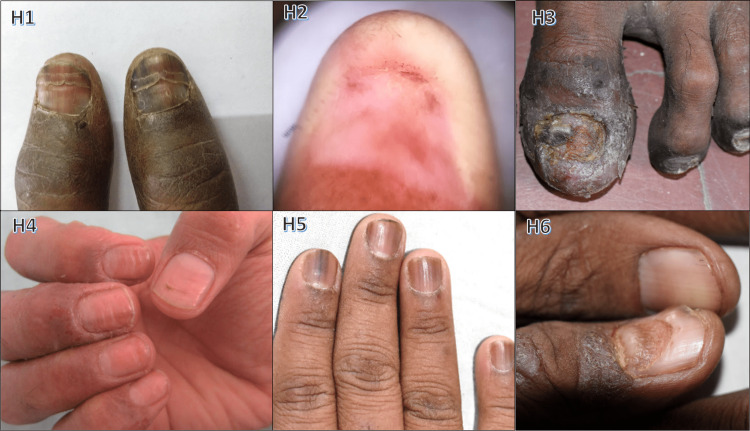
Drug-induced nail changes H1: doxorubicin-induced chromonychia; H2: carbamazepine-induced anonychia; H3: carboplatin-induced onychodystrophy; H4: cisplatin-induced Beau's lines; H5: zidovudine-induced melanonychia; H6: gemcitabine-induced onychomadesis

Among the cases, 13 (4.3%) were attributed to drug-induced nail changes (Figure 9). Notably, we observed specific patterns: five cases exhibited drug-induced nail plate discoloration (chromonychia), linked to docetaxel, doxorubicin, gemcitabine, and cyclophosphamide. Longitudinal melanonychia induced by zidovudine was seen in two HIV patients undergoing HAART. Additionally, two cases experienced temporary anonychia due to phenytoin and carbamazepine-induced toxic epidermal necrolysis. Gemcitabine led to onychomadesis in one case. Onycholysis occurred in one case due to docetaxel, while cisplatin caused multiple Beau’s lines and carboplatin induced onychodystrophy.

Less frequent nail changes observed were as follows - alopecia areata: five cases (1.67%), median Heller dystrophy: four cases (1.33%), racquet nail: three cases (1%), atopic dermatitis: three cases (1%), epidermolysis bullosa: three cases (1%), leprosy: three cases (1%), pityriasis rubra pilaris: two cases (0.67%), subungual warts: two cases (0.67%), vitiligo: two cases (0.67%), secondary syphilis: two cases (0.67%), pachyonychia congenita: two cases (0.67%). Also, a case each (0.33%) of the following conditions was found: Mee’s lines, onychoschisis, platynychia, triangular lunula, macrolunula, pincer nail, ingrown toenail, total leukonychia, and Koenen tumor.

**Figure 10:**
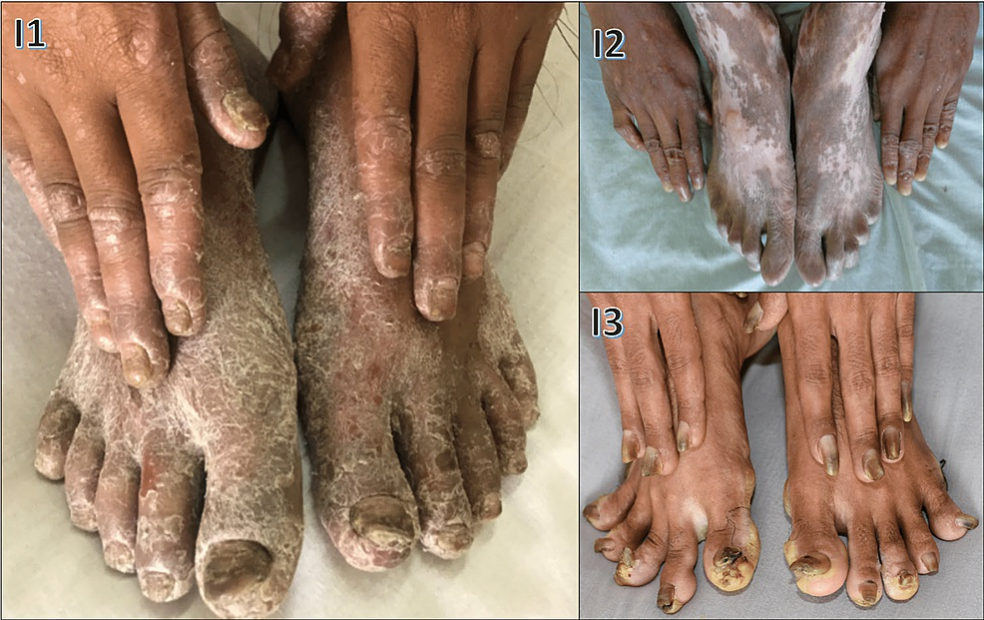
Various nail changes observed in our study - 1 I1: onycholysis, subungual hyperkeratosis, and thickening of the nail plate in pityriasis rubra pilaris; I2: loss of the nail plate in epidermolysis bullosa; I3: onychodystrophy in pachyonychia congenita

**Figure 11:**
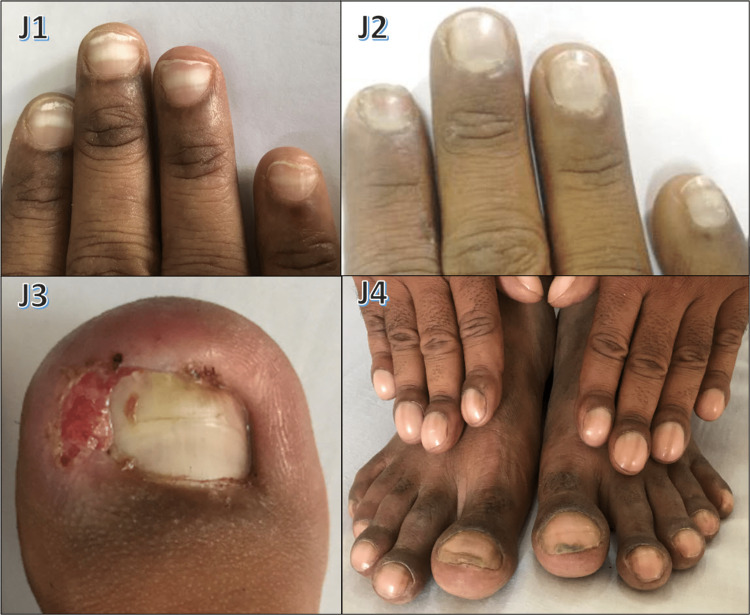
Various nail changes observed in our study - 2 J1: Mee's lines were seen in a chronic renal failure patient; J2: total idiopathic leukonychia; J3: ingrown toenail; J4: hereditary clubbing

**Figure 12:**
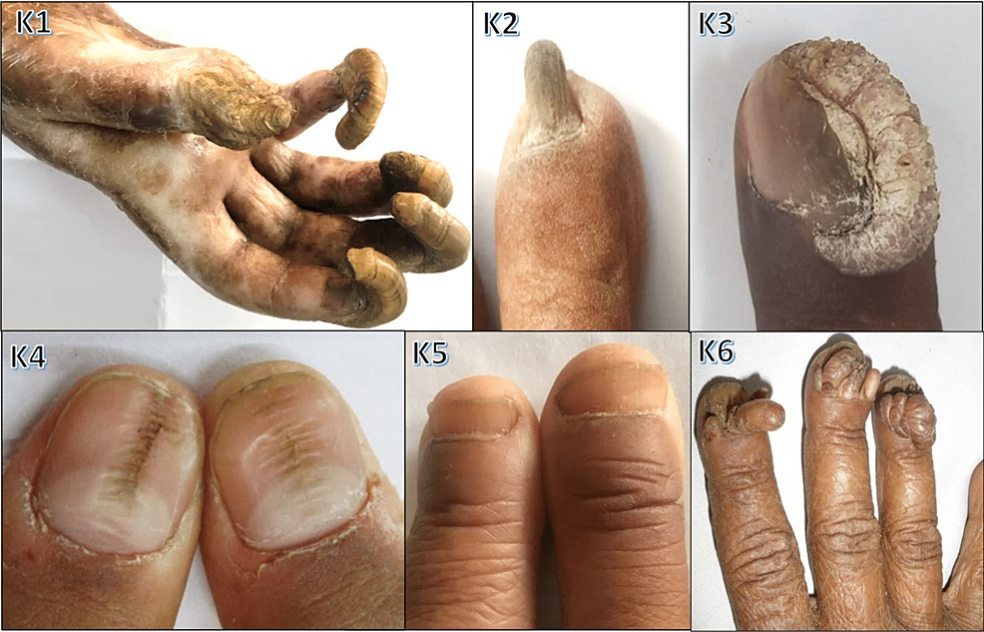
Various nail changes observed in our study - 3 K1: onychogryphosis in a post-burn patient; K2: pincer nail due to finger trauma; K3: periungual Wart; K4: median Heller dystrophy; K5: racquet nail; K6: Koenen tumor seen in tuberous sclerosis

**Figure 13:**
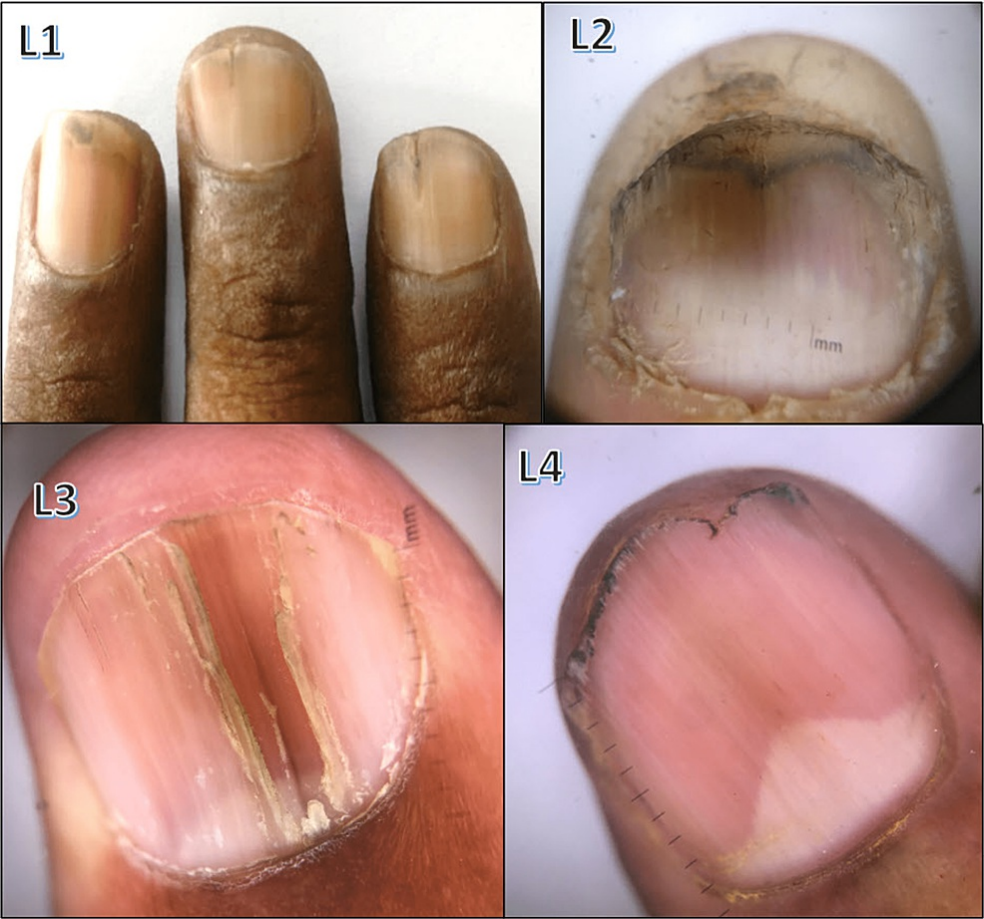
Various nail changes observed in our study - 4 L1: 'V'-shaped nicking in Darier's disease; L2: half-and-half nail seen in chronic renal failure; L3: onychoschizia; L4: triangular lunula seen in eczema

Various nail changes observed in pityriasis rubra pilaris, epidermolysis bullosa, pachyonychia congenita, Darier's disease, Mee's lines, half-and-half nails, ingrown nails, clubbing, total leukonychia, onychoschizia, triangular lunula, onychogryphosis, pincer nail, periungual wart, medial Heller dystrophy, racquet nail, and Koenen tumor are shown in Figures 10-13.

We have summarized the spectrum of nail changes observed in dermatological and systemic diseases in Table 8.

**Table 8:** Various patterns of nail changes observed in dermatological and systemic diseases CTD: connective tissue disease; HIV: human immunodeficiency virus; COPD: chronic obstructive pulmonary disease; SS: systemic sclerosis; SLE: systemic lupus erythematosus; DMS: dermatomyositis

Nail changes	Number of cases	Percentage	Associated diseases
Anonychia	3	1.00%	Trauma, drugs
Koilonychia	5	1.67%	Anemia
Platynychia	1	0.33%	Anemia
Thinning of the nail plate	7	2.33%	Lichen planus, trauma
Brittle nail	2	0.67%	Anemia
Pitting	67	22.33%	Psoriasis, eczema, alopecia areata, atopic dermatitis, secondary syphilis, pemphigus vulgaris, vitiligo
Longitudinal ridging	38	12.67%	Psoriasis, lichen planus, paronychia, alopecia areata, pemphigus vulgaris, CTD
Transverse ridging	5	1.67%	Atopic dermatitis, median Heller dystrophy
Splinter hemorrhage	7	2.33%	Psoriasis, Darier’s disease, pityriasis rubra pilaris, ischemic heart disease and hypertension, CTD
Salmon patch	7	2.33%	Psoriasis
Longitudinal melanonychia	12	4.00%	Lichen planus, drugs, HIV, idiopathic, Addison’s disease
Leukonychia	4	1.33%	Alopecia areata, Darier’s disease, idiopathic
Half-and-half nail	2	0.67%	Chronic renal failure
Triangular lunula	1	0.33%	Eczema
Red lunula	3	1.00%	Alopecia areata, CTD
Mee's line	1	0.33%	Chronic renal failure
Beau’s line	29	9.67%	Psoriasis, lichen planus, drugs, secondary syphilis, pemphigus vulgaris
Onychomadesis	8	2.67%	Alopecia areata, drugs, pemphigus vulgaris, epidermolysis bullosa, diabetes mellitus
Onychoschisis	1	0.33%	Trauma
Onycholysis	50	16.67%	Psoriasis, paronychia, eczema, pemphigus vulgaris, pityriasis rubra pilaris, drug-induced, leprosy, secondary syphilis
Pterigyum nail	3	1.00%	Lichen planus
Nicking	4	1.33%	Darier’s disease
Onychodystrophy	75	25.00%	Onychomycosis, psoriasis, lichen planus, paronychia, eczema, drugs, epidermolysis bullosa, pachyonychia congenita
Onychogryphosis	3	1.00%	Trauma, diabetes mellitus
Subungal hyperkeratosis	45	15.00%	Psoriasis, lichen planus, paronychia, eczema, atopic dermatitis, pemphigus vulgaris, Darier’s disease, pityriasis rubra pilaris
Nailfold erythema	52	17.33%	Onychomycosis, CTD
Nailfold telangiectasia	25	8.33%	CTD
Nailfold inflammation	17	5.67%	Paronychia, pemphigus vulgaris
Absent cuticle	15	5.00%	Paronychia
Pincer nail	1	0.33%	Trauma
Ingrown nail	1	0.33%	Trauma
Clubbing	5	1.67%	COPD, idiopathic, CTD, leprosy
Racquet nail	3	1.00%	Idiopathic
Transverse groove	13	4.33%	Psoriasis, paronychia
Ragged cuticle	9	3.00%	CTD (SS, SLE, DMS)
Macrolunula	5	1.67%	Habit tic, median Heller dystrophy
Nail plate discoloration	106	35.33%	Onychomycosis, psoriasis, paronychia, eczema, pemphigus vulgaris, Darier’s disease, pachyonychia congenita, pityriasis rubra pilaris, Addison’s disease, vitiligo

## Discussion

Nail disorders encompass a wide spectrum of conditions, spanning congenital, developmental, infectious, neoplastic, degenerative, dermatological, and systemic diseases. A comprehensive exploration of their clinical manifestations, incidence, and associations is crucial for precise diagnosis and effective management. In this study of 300 cases, the female-to-male ratio was 1.32:1 (Table 1). Drake et al. [3] found no significant sex-based distribution difference in nail disorders. The dominant age group in our study was 21-40 years (42%), followed by 41-60 years (28%) (Table 1). Nageswaramma et al. [4] observed a similar dominance of people aged 21-40 years in their study (40%). Onychomycosis (24.33%) (Figures 1, 2) was found to be the most common dermatosis with nail changes, followed by nail psoriasis (20%). Leyden and Kligman [5] indicated that onychomycosis is the most common infection and accounts for 20% of all nail disorders. Out of 24.33% cases of onychomycosis, it was seen mainly affecting females (57.5%) and housewives; the age group most affected was 21-40 years (46.57%), which aligns with the findings of Grover (56%) [6]. According to some other studies [6,7,8], DLSO incidence ranges from 64.44-90.5%, severe type TDO from 6-7.77%, with a low prevalence of onychomycosis (SWO). Our study showed the prevalence of DLSO at 65.7%, TDO at 12.32%, and a higher SWO rate (15.1%), possibly linked to increased HIV prevalence. Table 9 compares patterns of nail involvement in onychomycosis among different studies.

**Table 9:** Comparison of the pattern of nail involvement in onychomycosis among various studies DLSO: distal lateral subungual onychomycosis; PSO: proximal subungual onychomycosis; SWO: superficial white onychomycosis; TDO: total dystrophic onychomycosis

Morphological pattern	Current study	Grover [6]	Garg et al. [8]	Vinod et al. [7]
DLSO	65.7%	82%	64.44%	90.57%
PSO	6.8%	6%	4.44%	1.14%
SWO	15.1%	2%	1.11%	2.86%
TDO	12.32%	6%	17.77%	8.58%

Psoriasis, a common nail-affecting disease, causes dystrophy with pitting, onycholysis, subungual hyperkeratosis, splinter hemorrhages, and paronychia. Psoriatic nail changes (Figure 3) ranked second in our study (20%) in terms of prevalence, and was found primarily in the age group of 41-60 years. According to Ghosal et al. [9], pitting (90.23%) was the most common finding observed. In our study, pitting (88.3%) was the most common finding as well, followed by onycholysis and subungual hyperkeratosis. Puri and Kaur [10] also found pitting as the most common nail finding (70%), with Calvert et al. [11] noting pitting at a rate of 60%, onycholysis at 50%, and subungual hyperkeratosis at 45%. Our study had two psoriasis patients with diabetes, five with hypertension, and three with atopy, similar to the findings of Singh and Gupta [12]. Psoriatic arthritis, observed in 7% by Gladman et al. [13], occurred in 11.6% of cases in our study. Table 10 compares patterns of nail involvement in psoriasis among different studies.

**Table 10:** Comparison of the pattern of nail involvement in psoriasis among various studies

Nail changes in psoriasis	Current study	Tham et al. [14]	Puri and Kaur (Punjab study) [10]	Marina et al. [15]
Pitting	88.3%	67.5%	70%	67.34%
Subungual Hyperkeratosis	43.3%	24.7%	40%	39.14%
Onycholysis	56.6%	67.2%	52%	39.86%
Oil drop sign	11.7%	-	10%	39.14%
Beaus lines	33.3%	-	14%	-
Spinter Haemorrhage	3.3%	-	12%	22.57%
Dystrophy	10%	-	6%	-

Figure 4 illustrates nail changes in lichen planus. In this study, 11 cases (3.67%) showed lichen planus with nail involvement: six males and 5 females. The most commonly affected age group was 21-40 years, akin to the findings of Kanwar and De (30-40 years) [16]. Table 11 compares patterns of nail involvement in lichen planus between our study and the study by Puri and Kaur [10].

**Table 11:** Comparison of the pattern of nail involvement in lichen planus

Nail changes in lichen planus	Current study	Puri and Kaur [10]
Thinning of nail	54.5%	-
Longitudinal melanonychia	45.4%	20%
Trachyonychia	9.1%	8%
Longitudinal striations	63.6%	24%
Pterygium	27.2%	16%
Dystrophy	18.2%	4%
Beau’s line	36.4%	-

Figure 5 displays a male patient with twenty nail dystrophy (TND), also termed trachyonychia. TND affects all 20 nails uniformly and simultaneously, potentially idiopathic or linked to various disorders like lichen planus, psoriasis, etc. In our study, trachyonychia occurred in seven cases (2.33%), with 42.8% of them being idiopathic, 28.57% alopecia areata, and 14.28% attributed to lichen planus or psoriasis causes. Gordon et al. [17] and Garg et al. [18] reported idiopathic, lichen planus, and alopecia areata as common causes. Taniguchi et al. [19], Tosti et al. [20], and Jerasututus et al. [21] linked trachyonychia to alopecia areata, lichen planus, and psoriasis, suggesting an autoimmune involvement. Table 2 provides a comparison of trachyonychia-associated diseases among different studies.

**Table 12:** Comparison of trachyonychia-associated diseases among different studies

Trachyonychia-associated diseases	Current study	Puri and Kaur [10]	Garg et al. [18]
Alopecia areata	28.57%	10%	40%
Psoriasis	14.28%	25%	-
Lichen planus	14.28%	20%	20%
Idiopathic	42.8%	45%	40%

Nail involvement in pemphigus arises from bullous lesions in the nail bed/matrix or acantholysis of the lateral nail fold. In our study, pemphigus vulgaris showed paronychia (66.67%) and onychomadesis (50%) as the main nail findings (Figure 6). Habibi et al. [22] reported that 31.6% of pemphigus vulgaris patients had nail changes, with paronychia and onychomadesis being the more common ones. Engineer et al. [23] observed paronychia (60%) and onychomadesis (33%) in 15 pemphigus vulgaris patients. Cahali et al. [24] described five patients with onychomadesis and one with Beau's lines among nail-involved pemphigus cases.

Pachyonychia congenita, observed in two cases (0.67%) in our study, had childhood onset with palmoplantar keratoderma (Figure I3), follicular hyperkeratosis, and oral leukokeratosis. Sivasundaram et al. [25] noted autosomal dominant inheritance and variable expression. Jadassohn-Lewandowsky (PC-1) was the commonest type. In our study, pachyonychia congenita belonged to the Jadassohn-Lewandowsky type. Two females had pityriasis rubra pilaris; prevalent nail changes included thickening, onycholysis, and subungual hyperkeratosis (Figure I2), akin to the findings of Mortimer and Dawber [26]. The average number of nails affected was 11. Among six cases of Darier's disease, three cases(50%) showed distal subungual wedge-shaped hyperkeratosis, and four cases (66/7%) each had red and white longitudinal streaks and 'V'-shaped nicking, while splinter hemorrhages and leukonychia were observed in one case (16.6%) each. Histopathology confirmed Darier's. Zaias and Ackerman [27] reported nail changes in 73 Darier's patients, diagnostic with similar findings.

In our study, among 27 cases (9%) of connective tissue diseases (CTD), SLE (48.14%) was prominently linked to nail changes. Nail fold erythema (48.1%) followed by nail fold telangiectasis (44.4%) was seen more frequently. Nail fold erythema (61.5%) was common in SLE, and nail fold telangiectasia (80%) in SS. Scleroderma capillary pattern was found in 70% of SS cases, 66.7% of DMS cases, and 7.7% of SLE cases. NFC findings (scleroderma capillary pattern) were notably seen in Raynaud’s phenomenon patients. These results are consistent with those of Nabil et al. [28] and Elmansour et al. [29]. Of the 50 onycholysis cases (16.67%), the distribution of the various conditions was as follows - psoriasis: 34; paronychia: six; drug-induced: three; eczema and pityriasis rubra pilaris: two each; pemphigus vulgaris, secondary syphilis, and leprosy: one each. Ray et al. [30]^ ^highlighted the role of local factors in onycholysis over systemic causes.

Pitting was seen in 67 cases (22.33%) in our study, with psoriasis being the most common cause (79.1%) followed by eczema (7.5%), alopecia areata (5.9%), and atopic dermatitis (1.5%). Puri and Kaur [10] found pitting as the most common manifestation of psoriasis (70%). Zaias and Ackerman noted pitting in chronic eczematous dermatitis, alopecia areata, and even without any apparent disease. Onychogryphosis was noted in three cases (1%), 66.67% due to trauma, both involving great toenails and one case was seen involving fingernails secondary to burn injury (Figure 11). Gilchrist [31] and Cohen et al. [32] describe it to be an acquired dystrophy often in great toenails, common in the elderly, while trauma or foot issues may trigger it in middle age. Clubbing was found in five cases (1.67%): idiopathic (one case, Figure 11 J4), COPD (1), and three cases (1%) with clubbing and finger resorption - systemic sclerosis (1) and leprosy (2). Dawber and Baran [33] cite thoracic organ disorders (80%) and alimentary tract (5%), endocrine, and idiopathic issues as causes. Tosti et al. [34] noted osteolysis and bone telescoping in leprosy, and connected parrot's beak nail to severe acrosclerosis in SS.

In our study, drug-induced nail changes were present in 13 cases (4.3%) (Figure 9). The primary chemotherapy-related alteration was nail plate discoloration, seen in three cases (38.46%). Similar findings were reported by Zawar et al. [35], who observed chromonychia (54.26%) and nail dystrophy (29.45%) as common chemotherapy-related changes. Onychodystrophy due to carboplatin and onychomadesis from gemcitabine were each seen once (Figure 9, H3 and H6), while docetaxel caused onycholysis. Saini et al. [36] noted onycholysis, Beau's lines, onychomadesis, pyogenic granuloma, and paronychia in taxane-treated patients. Doxorubicin contributed to longitudinal pigmented bands and nail discoloration (Figure 9, H1). Zidovudine was linked to longitudinal melanonychia in two cases (Figure 9, H4), consistent with the findings of Cribier et al.'s [37] study on HIV-positive patients, where longitudinal melanonychia was prevalent in 14.8% of cases. Subungual warts were found in one case (0.33%), associated with verruca vulgaris (Figure 12, K3). De Berker et al. [1] observed that subungual warts were the most common tumor involving nails. Subungual and periungual warts, mildly contagious, likely result from HPV DNA entry through skin biting or pricking.

One case (0.33%) involved an ingrowing toenail (Figure 11, J3). Cambiaghi et al.'s [38] study showed that the main cause of ingrown nails is compression of toes from the side due to ill-fitting footwear and the main contributory cause is cutting the toenail in a half circle instead of straight across. Eight cases (2.67%) of onychomadesis were seen in our study, three of which were linked to pemphigus vulgaris (Figure 6, E1 and E2). Habibi et al. reported that 31.6% of pemphigus vulgaris patients had nail changes, with paronychia and onychomadesis being the most common [22]. Macrolunula was observed in five cases (1.67%): four linked to median Heller dystrophy (Figure 12, K4) and one to habit tic. Cohen [39] noted that macrolunula is a normal variant in India and is tied to nail disorders (habit tic, median nail dystrophy, hyperthyroidism). Twelve cases (4%) of longitudinal melanonychia were seen: two of them idiopathic and four HIV-related. Cribier et al. [37]^ ^highlighted its prevalence in HIV-positive individuals, possibly due to HAART or disease. Nail symptoms were found more commonly in HIV patients than healthy controls, possibly linked to immunosuppression levels.

According to Collins [40], longitudinal melanonychia often manifests a racial (Afro-Caribbean) variation; 77% of people over 20 years of age had it, which went up to almost 100% by age 50 years. Koilonychia was seen in five cases (1.67%), due to Iron deficiency anemia (Figure 7, F3). Bergaron et al. [41] cite hereditary, acquired, and idiopathic factors; hypochromic iron deficiency was the most frequent cause. Idiopathic total leukonychia was seen in one case (0.33%), which was of childhood onset, with no family history (Figure 11, J2). Grossman and Scher [42] reported an autosomal dominant trait with variable penetrance in a black family with total leukonychia occurring across three generations, despite having no other abnormalities. Transverse grooves in nails were found in 13 cases (4.33%), due to psoriasis and paronychia. Macaulay [43] noted grooves from isolated diseases, trauma, inflammation, and neurology. Two cases (0.67%) of half-and-half nails in chronic renal failure on dialysis were observed (Figure 13, L2). Lindsay [44]^ ^found 84% of cases linked to azotemia, 8-9% to cylindriuria without azotemia, and 4% to reduced creatinine clearance without azotemia. Anonychia was seen in three cases (1%): two due to drug-induced toxic epidermal necrolysis (Figure 9, H2) and one local trauma. Dawber and Baran [33] and Telfer et al. [45] noted congenital or transient origins due to nail loss from local/systemic causes. Racquet nails were seen in three male patients (1%), involving all fingernails (Figure 12, K5). Dawber and Baran [33] noted that autosomal dominant inheritance, common in girls, may affect any finger but often the thumb. One case (0.33%) of pincer nails due to trauma was seen (Figure 12, K2). Baran et al. [46]^ ^stated that pincer nails are linked to ill-fitting shoes, trauma, and osteoarthritis.

Limitations of the study

The study was conducted at a single tertiary care hospital in Ahmedabad, and hence the findings may not be representative of the whole population of India or other countries. The sample size of the study was relatively small, which may limit the generalizability of the findings. The study did not include a control group, which may limit the ability to draw conclusions about the prevalence of nail disorders in the general population. Moreover, the study relied on clinical examination and laboratory investigations, which may not be sufficient to diagnose all types of nail disorders. Finally, the study did not assess the impact of nail disorders on the quality of life of the patients.

## Conclusions

Recognizing and describing nail findings accurately are crucial for diagnosing nail disorders. Abnormal nails hold paramount clinical significance, especially when they are an exclusive feature without other apparent disease symptoms. This study sheds light on the pivotal role nails play in reflecting underlying health conditions, emphasizing the need for meticulous nail examination in clinical practice. These findings offer clues to diagnose systemic diseases, making nails a valuable diagnostic tool. Given the scarcity of studies on nail disorders, this research unveils the spectrum of nail disorders and their associations. We believe our findings offer a deeper understanding of these often-overlooked aspects of healthcare. The study also underscores the enduring importance of physical findings in the contemporary landscape of diagnostic tests and procedures, highlighting the role of bedside medicine.

## References

[1] De Berker DAR, Baran R, Dawber RPR (2004). Disorders of nails. Rooks Textbook of Dermatology, 7th Edition.

[2] Myers KA, Farquhar DR (2001). The rational clinical examination. Does this patient have clubbing?. JAMA.

[3] (1996). Guidelines of care for nail disorders. American Academy of Dermatology. J Am Acad Dermatol.

[4] Nageswaramma S, Kumari DG, Vani T, Ragini P, Glory DG (2016). A clinico-epidemiological study of nail changes in various dermatoses. IOSR J Dent Med Sci.

[5] Leyden JJ, Kligman AM (1978). Interdigital athlete's foot. The interaction of dermatophytes and resident bacteria. Arch Dermatol.

[6] Grover S (2003). Clinico-mycological evaluation of onychomycosis at Bangalore and Jorhat. Indian J Dermatol Venereol Leprol.

[7] Vinod S, Grover S, Dash K, Singh G (2000). A clinico - mycological evaluation of onychomycosis. Indian J Dermatol Venereol Leprol.

[8] Garg A, Venkatesh V, Singh M, Pathak KP, Kaushal GP, Agrawal SK (2004). Onychomycosis in central India: a clinicoetiologic correlation. Int J Dermatol.

[9] Ghosal A, Gangopadhyay DN, Chanda M, Das NK (2004). Study of nail changes in psoriasis. Indian J Dermatol.

[10] Puri N, Kaur T (2012). A study of nail changes in various dermatosis in Punjab, India. Our Dermatol Online.

[11] Calvert HT, Smith MA, Wells RS (1963). Psoriasis and the nails. Br J Dermatol.

[12] Singh MK, Gupta SK (2015). Demographic study of psoriasis in eastern Uttar Pradesh India. J Evol Med Dent Sci.

[13] Gladman DD, Antoni C, Mease P, Clegg DO, Nash P (2005). Psoriatic arthritis: epidemiology, clinical features, course, and outcome. Ann Rheum Dis.

[14] Tham SN, Lim JJ, Tay SH, Chiew YF, Chua TN, Tan E, Tan T (1988). Clinical observations on nail changes in psoriasis. Ann Acad Med Singap.

[15] Marina EM, Botar-Jid C, Bolboaca SD, Roman II, Senila CS, Mihu CM, Tataru DA (2017). Patterns of clinical nail appearances in patients with cutaneous psoriasis. Clujul Med.

[16] Kanwar AJ, De D (2010). Lichen planus in childhood: report of 100 cases. Clin Exp Dermatol.

[17] Gordon KA, Vega JM, Tosti A (2011). Trachyonychia: a comprehensive review. Indian J Dermatol Venereol Leprol.

[18] Garg P, Kumar A, Rathore PK, Goyal S (2017). Clinical study of various nail disorders presenting to dermatology outpatient department. Int J Adv Integr Med Sci.

[19] Taniguchi S, Kutsuna H, Tani Y, Kawahira K, Hamada T (1995). Twenty-nail dystrophy (trachyonychia) caused by lichen planus in a patient with alopecia universalis and ichthyosis vulgaris. J Am Acad Dermatol.

[20] Tosti A, Bardazzi F, Piraccini BM, Fanti PA (1994). Idiopathic trachyonychia (twenty-nail dystrophy): a pathological study of 23 patients. Br J Dermatol.

[21] Jerasutus S, Suvanprakorn P, Kitchawengkul O (1990). Twenty-nail dystrophy: a clinical manifestation of spongiotic inflammation of the nail matrix. Arch Dermatol.

[22] Habibi M, Mortazavi H, Shadianloo S (2008). Nail changes in pemphigus vulgaris. Int J Dermatol.

[23] Engineer L, Norton LA, Ahmed AR (2000). Nail involvement in pemphigus vulgaris. J Am Acad Dermatol.

[24] Cahali JB, Kakuda EY, Santi CG, Maruta CW (2002). Nail manifestations in pemphigus vulgaris. Rev Hosp Clin Fac Med Sao Paulo.

[25] Sivasundram A, Rajagopalan K, Sarojini T (1985). Pachyonychia congenita. Int J Dermatol.

[26] Mortimer PS, Dawber RP (1985). Dermatologic diseases of the nail unit other than psoriasis and lichen planus. Dermatol Clin.

[27] Zaias N, Ackerman AB (1973). The nail in Darier-White disease. Arch Dermatol.

[28] Nabil PA, Rao R, Shenoi SD, Balachandran C (2006). Nail unit in collagen vascular diseases: a clinical, histopathological and direct immunofluorescence study. Indian J Dermatol.

[29] Elmansour I, Chiheb S, Benchikhi H (2014). Nail changes in connective tissue diseases: a study of 39 cases. Pan Afr Med J.

[30] Navarro L (2023). Pattern diagnosis of onycholysis. JEADV Clin Pract.

[31] Gilchrist AK (1979). Common foot problems in the elderly. Geriatrics.

[32] Cohen PR, Scher RK (1992). Geriatric nail disorders: diagnosis and treatment. J Am Acad Dermatol.

[33] (1994). Diseases of the Nails and Their Management. Blackwell Scientific Publications.

[34] Tosti A, Baran R, Dawber RP (2001). The nail in systemic diseases and drug‐induced changes. Baran and Dawber's Diseases of the Nails and Their Management.

[35] Zawar V, Bondarde S, Pawar M, Sankalecha S (2019). Nail changes due to chemotherapy: a prospective observational study of 129 patients. J Eur Acad Dermatol Venereol.

[36] Saini K, Sutaria A, Shah B, Brahmbhatt V, Parmar K (2019). Cutaneous adverse drug reactions to targeted chemotherapeutic drugs: a clinico-epidemiological study. Indian J Dermatol.

[37] Cribier B, Mena ML, Rey D, Partisani M, Fabien V, Lang JM, Grosshans E (1998). Nail changes in patients infected with human immunodeficiency virus. A prospective controlled study. Arch Dermatol.

[38] Cambiaghi S, Pistritto G, Gelmetti C (1997). Congenital hypertrophy of the lateral nail folds of the hallux in twins. Br J Dermatol.

[39] Cohen PR (1996). The lunula. J Am Acad Dermatol.

[40] Collins RJ (1984). Melanomas in the Chinese among southwestern Indians. Cancer.

[41] Baran R, Dawber RP, Richert B (2001). Physical signs. Baran and Dawber’s diseases of the nails and their management.

[42] Grossman M, Scher RK (1990). Leukonychia. Review and classification. Int J Dermatol.

[43] Macaulay WL (1966). Transverse ridging of the thumbnails. "Washboard thumbnails". Arch Dermatol.

[44] Lindsay PG (1967). The half-and-half nail. Arch Intern Med.

[45] Telfer NR, Barth JH, Dawber RP (1988). Congenital and hereditary nail dystrophies--an embryological approach to classification. Clin Exp Dermatol.

[46] Baran R, Haneke E, Richert B (2001). Pincer nails: definition and surgical treatment. Dermatol Surg.

